# Digital gene expression analysis in mice lung with coinfection of influenza and streptococcus pneumoniae

**DOI:** 10.18632/oncotarget.23104

**Published:** 2017-12-11

**Authors:** Jun Luo, Linlin Zhou, Hongren Wang, Zhen Qin, Li Xiang, Jie Zhu, Xiaojun Huang, Yuan Yang, Wanyi Li, Baoning Wang, Mingyuan Li

**Affiliations:** ^1^ Department of Microbiology, West China School of Basic Medical Sciences and Forensic Medicine, Sichuan University, Chengdu 610041, China; ^2^ Department of Microbiology, Guizhou Medical University, Guiyang 550004, China; ^3^ State Key Laboratory of Oral Diseases, Sichuan University, Chengdu, Sichuan 610041, China

**Keywords:** influenza A virus, streptococcus pneumoniae, digital gene expression, apoptosis

## Abstract

Influenza A virus (IAV) and Streptococcus pneumoniae (SP) are two major upper respiratory tract pathogens that can also cause infection in polarized bronchial epithelial cells to exacerbate disease in coinfected individuals which may result in significant morbidity. However, the underlying molecular mechanism is poorly understood. Here, we employed BALB/c ByJ mice inflected with SP, IAV, IAV followed by SP (IAV+SP) and PBS (Control) as models to survey the global gene expression using digital gene expression (DGE) profiling. We attempt to gain insights into the underlying genetic basis of this synergy at the expression level. Gene expression profiles were obtain using the Illimina/Hisseq sequencing technique, and further analyzed by enrichment analysis of Gene Ontology (GO) and Pathway function. The hematoxylin-eosin (HE) staining revealed different tissue changes in groups during which IAV+SP group showed the most severe cell apoptosis. Compared with Control, a total of 2731, 3221 and 3946 differentially expressed genes (DEGs) were detected in SP, IAV and IAV+SP respectively. Besides, sixty-two GO terms were identified by Gene Ontology functional enrichment analysis, such as cell killing, biological regulation, response to stimulus, signaling, biological adhesion, enzyme regulator activity, receptor regulator activity and translation regulator activity. Pathway significant enrichment analysis indicated the dysregulation of multiple pathways, including apoptosis pathway. Among these, five selected genes were further verified by quantitative reverse transcription-polymerase chain reaction (qRT-PCR). This study shows that infection with SP, IAV or IAV+SP induces apoptosis with different degrees which might provide insights into the molecular mechanisms to facilitate further research.

## INTRODUCTION

Secondary bacterial pneumonia is an important complication responsible for illness and death during epidemic and pandemic influenza [1]. A number of causative bacteria have been described in patients, including *Staphylococcus aureus* and *Haemophilus influenza*, but SP is the most common pathogen, and over 50% of the deaths have been attributed to secondary pneumococcal pneumonia following primary influenza virus infection [2]. The highly variable IAV evolve rapidly and cause epidemics and pandemic of acute respiratory disease that are often characterized by high morbidity and mortality in humans [3]. Secondary SP infections increase the severity and lethality in influenza virus-infected humans based on co-pathogenesis of both pathogens, also called lethal synergismas reviewed recently [4]. Analysis of the host response in the lungs of mice during 1918 virus infection revealed dramatically alveolar epithelial damage and persisted unabated until death [5]. Similar studies in ferrets and cynomolgus macaques demonstrated that the 1918 virus was highly lethal in both species and with severe lung pathology similar to that seen in mice [6, 7]. These studies revealed that the reconstructed 1918 pandemic influenza virus was highly pathogenic in several animal models.

Secondary bacterial pneumonia is still a significant cause of morbidity and mortality in years where the viruses are not virulent enough to cause severe lung damage or death from the virus alone [8–10]. However, the possible mechanisms of underlying the association between influenza and the following pneumococcal infections are still unclear, exploration for the underlying mechanisms would be a research priority because of their potentially important implications for prevention and control.

Recent studies have reported some genes might relate to cell apoptosis after coinfected with virus and bacteria, however, to the best of our knowledge, no study has been reported on the coinfection of SP and IAV in a comprehensive genetic view.

The digital gene expression (DGE) system is always used to do the comparative gene expression research which is a tag-based transcriptome sequencing approach. When using DGE, clean reads with certain end nuclease were strained through raw reads, and then mapped to the corresponding reference database. The quantity of each mRNA yielded from each gene are defined by the expression degree of the actual total genes in each sample [11]. In our study, we conducted a massive analysis of gene expression changes by using DGE when mice were infected with SP, IAV or IAV+SP, to better understand how the coinfection caused high morbidity and mortality in mice.

## OBJECTIVE

The objective of this study is to construct mice model of the coinfection of IAV+SP and to apply high throughput sequencing method to acquire gene expression changes of transcriptome in mice lung tissues infecting with IAV and secondary SP, which helps to perfect the molecular mechanism of the coinfection and provides research clues and ideas to further clarify the mechanism of the coinfection.

## RESULTS

### Lethal synergism

**Figure 1:**
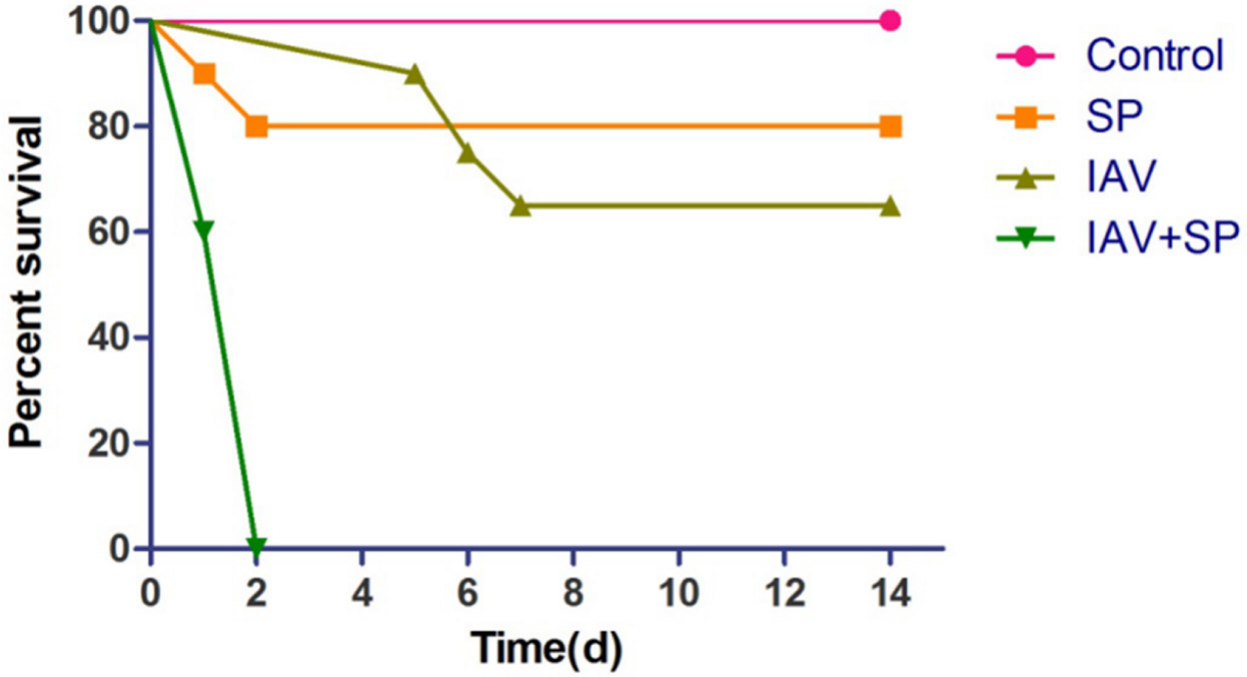
Synergistic mortality Groups of mice (*n* = 20) were infected with either SP, IAV or IAV+SP.

**Figure 2:**
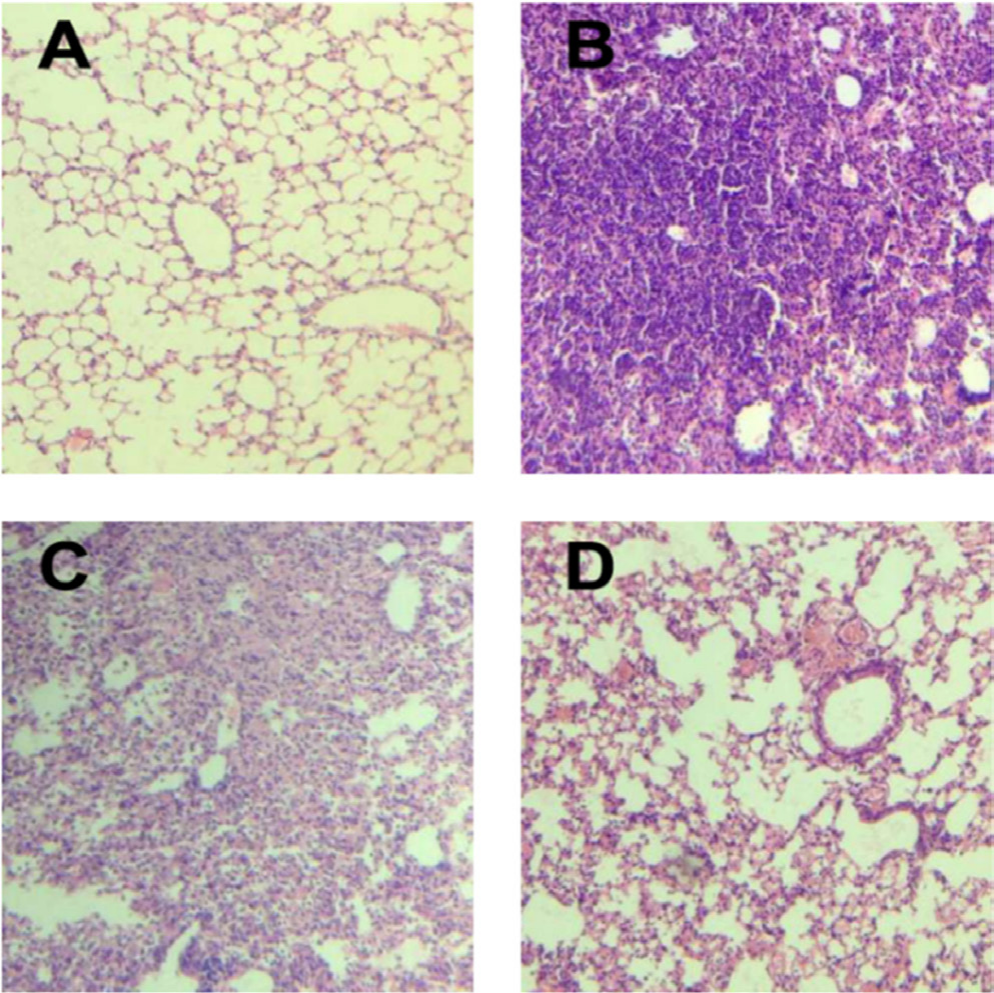
Histopathological changes in lung tissues with HE staining (100× magnification) (**A**) HE staining of lung tissue in control group; (**B**) HE staining of lung tissue in IAV+SP group; (**C**) HE staining of lung tissue in IAV group; (**D**) HE staining of lung tissue in SP group; HE: hematoxylin-eosin; IAV: Influenza A virus; SP: Streptococcus pneumoniae.

**Figure 3:**
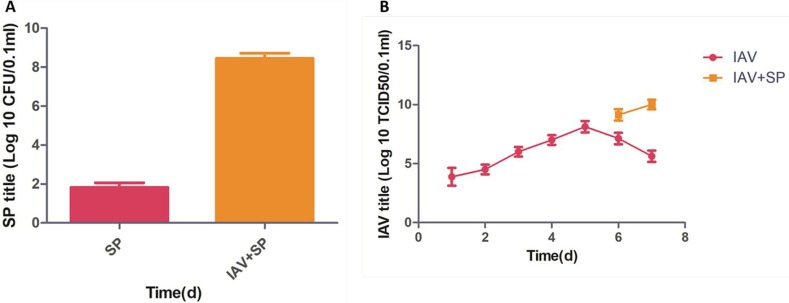
Titer changes after infections of SP, IAV and IAV+SP (**A**-**B**).

Mice infected with either SP or IAV alone exhibited mortality rates of 20% and 35%, respectively. All mice infected with IAV+SP succumbed to infection in <48 h (*P* < 0.0001, for difference in survival, vs. all other groups), demonstrating that synergistic mortality occurs in the model (Figure 1). Lung tissues in mice were analyzed by hematoxylin-eosin (HE) staining. Normal lung tissues could be observed in mice from control (Figure 2A). For tissues from IAV+SP group, severely adverse histopathology changes were observed which revealed large amount of infiltrated inflammatory cell, consolidations of lung tissue, and disappearances of pulmonary alveoli (Figure 2B). While histopathology lung tissues from IAV and SP groups showed moderately (Figure 2C) and mildly (Figure 2D) adverse changes, respectively. Titers of SP or IAV in lung tissues was measured after infection. Titer of SP from IAV+SP group was found to be significantly higher than that from SP group after 48 hours of infection (Figure 3A). Titer of IAV in IAV group reached a peak in five days of infection, but dramatically increased IAV titer could be observed once infected with SP afterwards (Figure 3B).

### Analysis of DGE libraries

**Figure 4:**
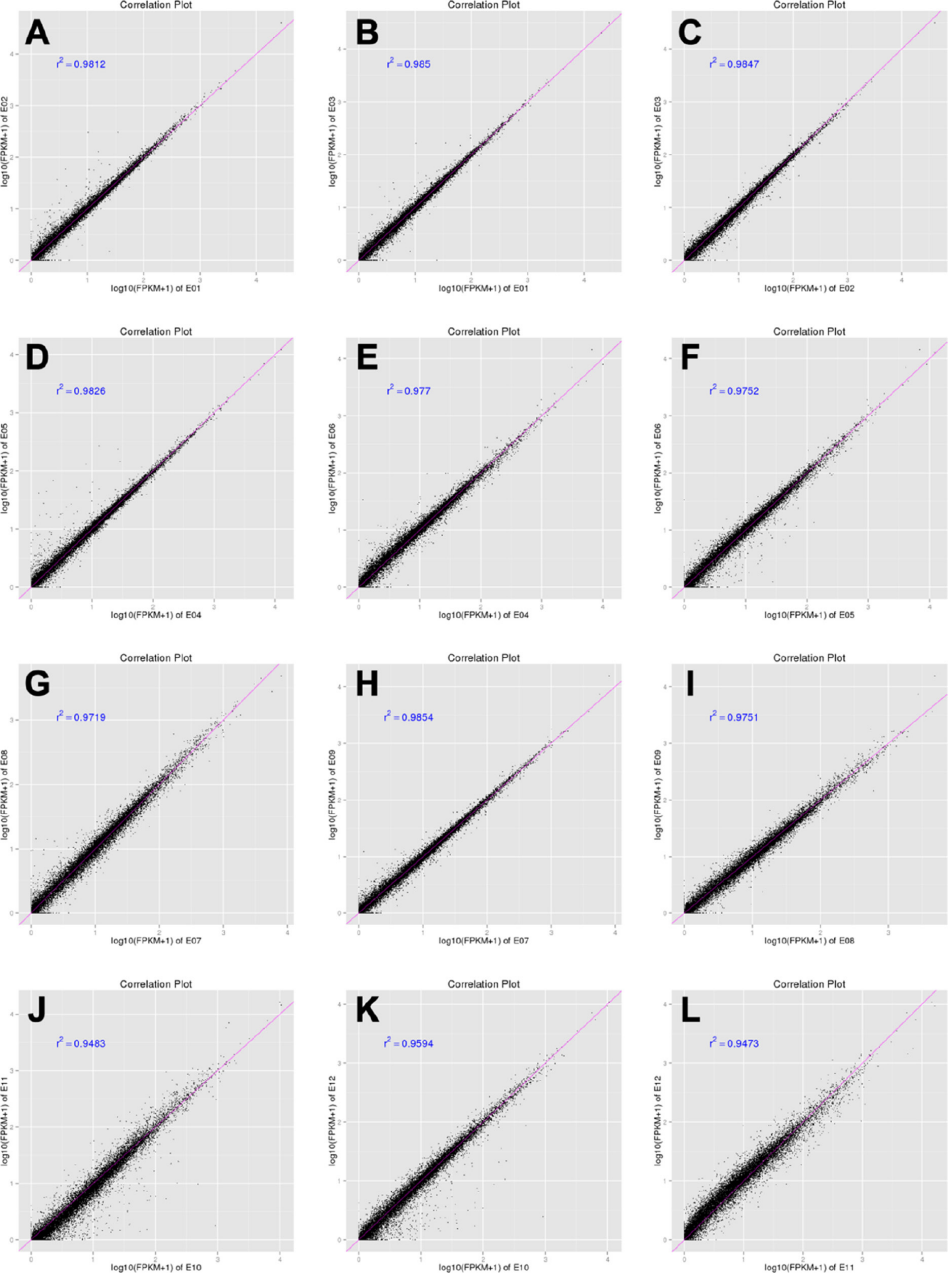
Correlation tests for the replicates The abscissa represents the value log10 (FPKM + 1) of one duplicate; the ordinate represents the value log10 (FPKM + 1) of the other duplicate. r^2^ is the square of Pearson Correlation Coefficient. E01,E02,E03 represent the three biological replicates of Control (**A**, **B**, **C**); E04,E05,E06 represent the three biological replicates of SP (**D**, **E**, **F**); E07,E08,E09 represent the three biological replicates of IAV (**G**, **H**, **I**); E01,E02,E03 represent the three biological replicates of IAV+SP (**J**, **K**, **L**).

**Figure 5:**
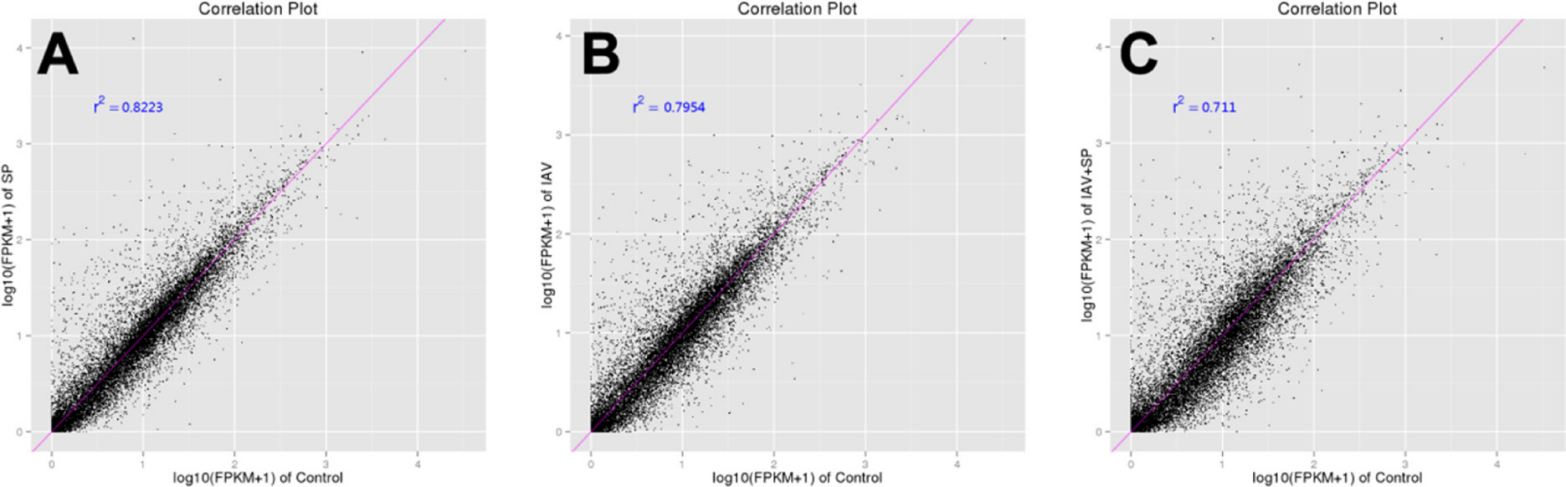
Correlation tests for groups of different treatments The abscissa represents the value log10 (FPKM + 1) of one group; the ordinate represents the value log10 (FPKM + 1) of the other group. r^2^ is the square of Pearson Correlation Coefficient. (**A**) Control VS SP; (**B**) Control VS IAV; (**C**) Control VS IAV+SP.

A total of twelve RNA samples, generated from three biological replicates under the four treatments were subjected to Using Illumina Hiseq2500 sequencing (Table 1). Approximately 11.4–13.4 million raw reads were sequenced per sample. After filtering out low-quality data (reads containing unknown base N and only adaptor reads), approximately 11.0–13.0 million clean reads remained in each library. The Q30 values (sequencing error rate, 0.1%) were more than 93.04%, and the GC percentages were between 52.89–53.11%. Approximately 87.96–91.06% reads in the twelve DGE libraries were unique mapped reads, suggesting that the profiling was reliable. To evaluate the reproducibility of DEG library sequencing, a Pearson correlation analysis was performed for every three replicates. The square of the Pearson correlation coefficient (*r*^2^) was greater than 0.94, indicating both operational stability and the reliability of the experimental results (Figure 4). For Control vs SP, Control vs IAV and Control vs IAV+SP, the Pearson correlation coefficient was 0.822, 0.795, 0.711, respectively, suggesting that the SP+IAV induced the most changes of gene expression profile which should be related to the pathogenesis of mice lung tissue (Figure 5).

**Table 1:** Summary for DGE sequencing dataset

Sample name	Raw reads	Clean reads	Total bases	Mapped reads	Uniq mapped reads	Multiple Map reads	Reads map to ‘+’	Reads map to ‘–’	GC content	% ≥Q30
Control_1	13,436,180	13059967	665976339	12,245,594 (93.76%)	11,892,783 (91.06%)	352,811 (2.70%)	6,051,986 (46.34%)	6,067,065 (46.46%)	53.05%	0.94
Control_2	11652141	11346855	578615876	10,620,247 (93.60%)	10,294,003 (90.72%)	326,244 (2.88%)	5,244,975 (46.22%)	5,250,388 (46.27%)	53.11%	0.94
Control_3	12573873	12182826	621246811	11,369,053 (93.32%)	11,018,016 (90.44%)	351,037 (2.88%)	5,613,900 (46.08%)	5,622,666 (46.15%)	52.89%	0.93
SP_1	12210156	11843851	603959314	11,018,282 (93.03%)	10,619,693 (89.66%)	398,589 (3.37%)	5,421,665 (45.78%)	5,436,527 (45.90%)	52.95%	0.94
SP_2	12025104	11670363	595113122	10,801,886 (92.56%)	10,410,413 (89.20%)	391,473 (3.35%)	5,310,270 (45.50%)	5,326,496 (45.64%)	53.02%	0.94
SP_3	11769692	11418955	582292359	10,583,344 (92.68%)	10,215,301 (89.46%)	368,043 (3.22%)	5,206,586 (45.60%)	5,220,412 (45.72%)	52.91%	0.93
IAV_1	12696076	12297419	627087395	11,358,377 (92.36%)	10,984,142 (89.32%)	374,235 (3.04%)	5,601,490 (45.55%)	5,614,813 (45.66%)	52.91%	0.94
IAV_2	13262578	12866027	656082743	11,789,646 (91.63%)	11,317,209 (87.96%)	472,437 (3.67%)	5,776,515 (44.90%)	5,804,632 (45.12%)	53.11%	0.93
IAV_3	13396497	12983885	662092858	12,094,096 (93.15%)	11,705,196 (90.15%)	388,900 (3.00%)	5,966,244 (45.95%)	5,978,450 (46.05%)	52.97%	0.94
SP+IAV_1	12582446	12188615	621537587	11,205,000 (91.93%)	10,783,215 (88.47%)	421,785 (3.46%)	5,505,381 (45.17%)	5,529,443 (45.37%)	53.10%	0.93
SP+IAV_2	11428961	11080378	565024547	10,234,359 (92.36%)	9,851,073 (88.91%)	383,286 (3.46%)	5,035,079 (45.44%)	5,045,563 (45.54%)	52.91%	0.93
SP+IAV_3	12423682	12016185	612745601	11,039,713 (91.87%)	10,626,010 (88.43%)	413,703 (3.44%)	5,425,391 (45.15%)	5,444,278 (45.31%)	53.10%	0.94

### Analysis of GO and KEGG pathways

**Figure 6:**
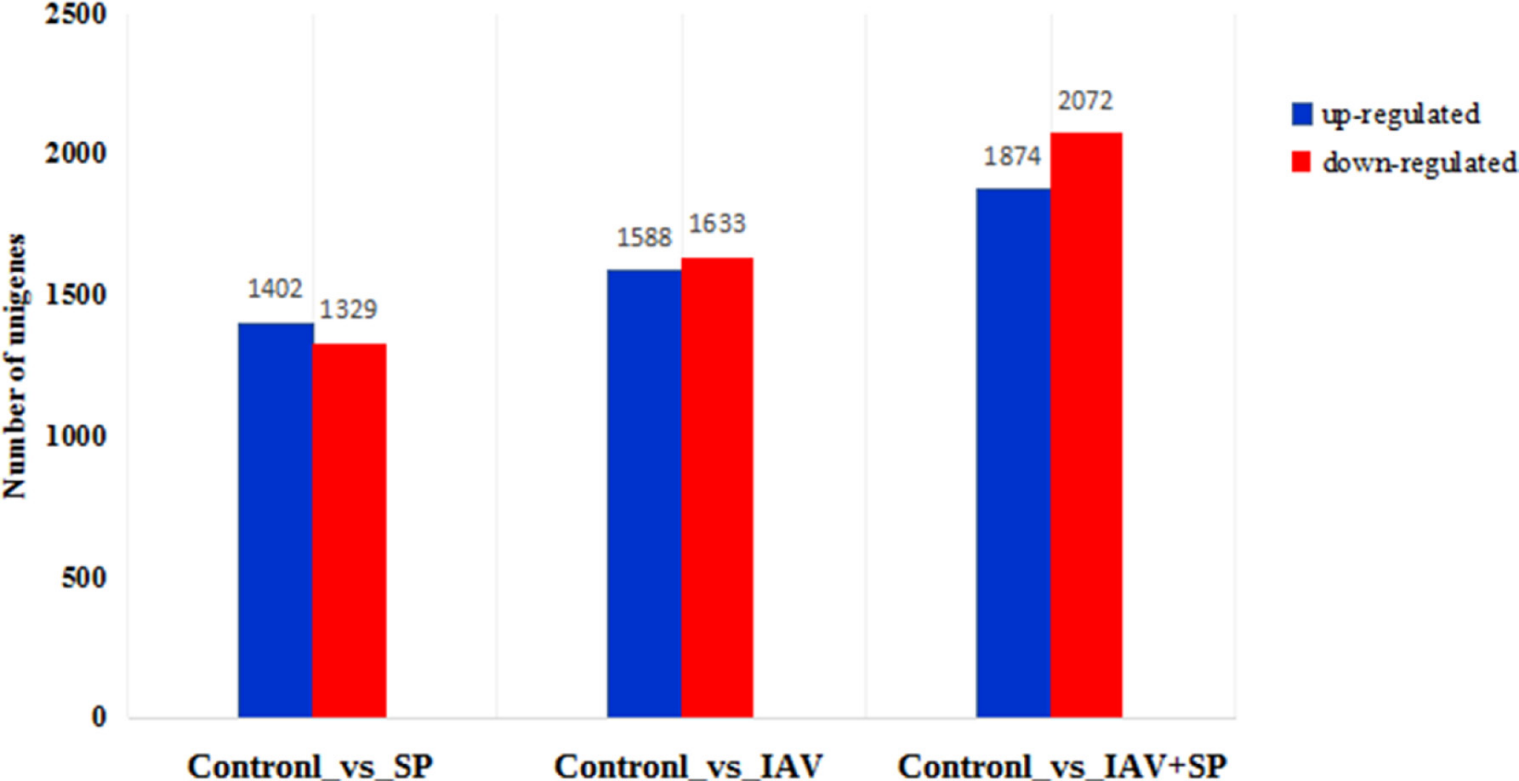
Number of DEGs in different groups The abscissa represents the three groups; the ordinate represents the number of unigenes.

Results showed that 2731, 3221, 3946 DEGs were detected in Control vs SP, Control vs IAV and Control vs SP+IAV comparisons respectively, of which, 1402, 1588 and 1874 were up-regulated and 1329, 1633 and 2072 were down-regulated in the three comparisons (Figure 6).

**Figure 7:**
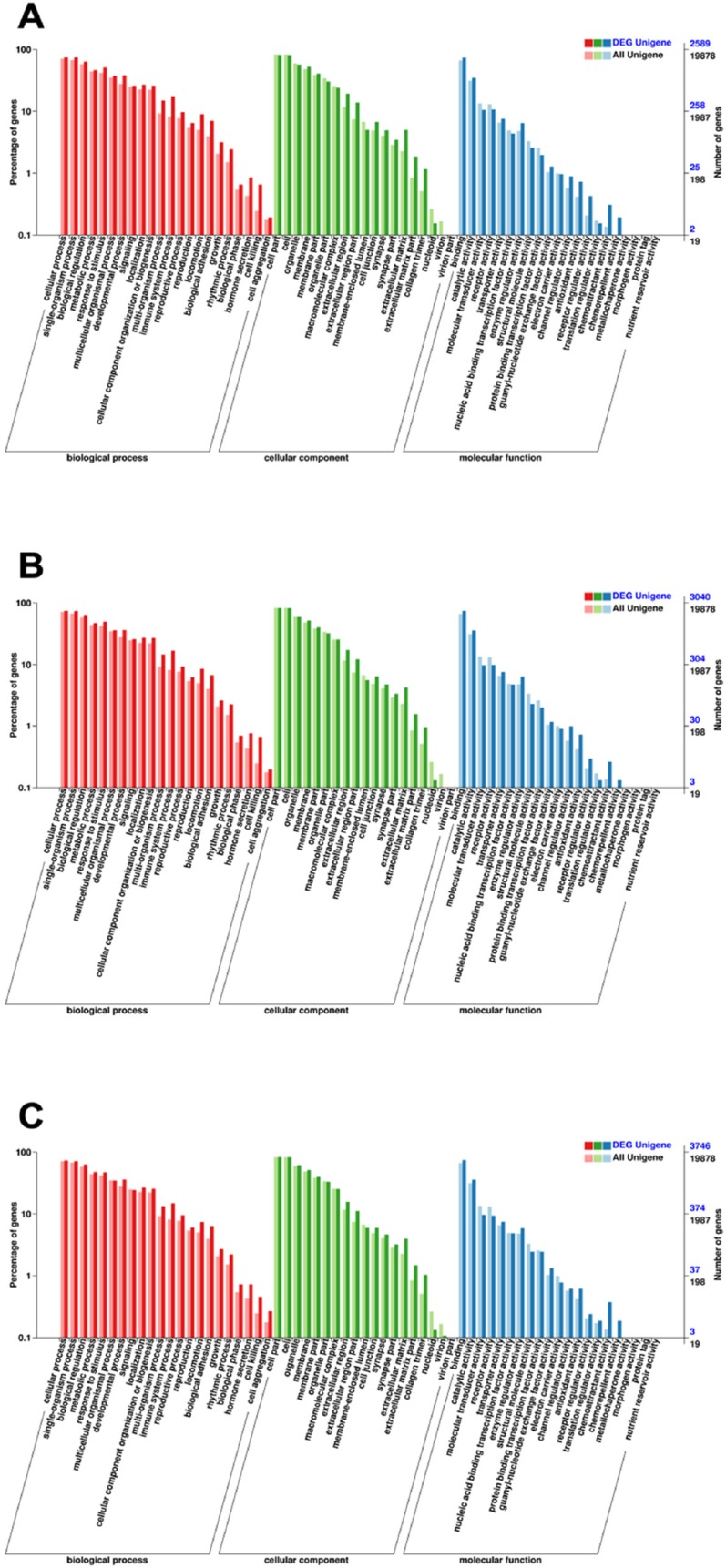
Categories of DEGs based on GO function The results are summarized in three major categories: biological process, cellular component and molecular function. The abscissa represents the Categories in GO database; the ordinate represents the percentage of genes. (**A**) Categories of DEGs of SP. (**B**) Categories of DEGs of IAV. (**C**) Categories of DEGs of IAV+SP.

To understand their functions, all the DEGs were mapped to terms of GO database, and then the DEGs were compared with the whole reference database. There are three ontologies in GO database, namely, molecular function, cellular component and biological process which are further subdivided into 62 subcategories in our study. The biological process contains 22 categories, cellular component contains 19 categories and molecular function contains 21 categories. The subcategory at the “cell part,” “cell,” “cellular process,” “single-organism process,” “binding,” “biological regulation,” “organelle, ”“membrane,” “response to stimulus,” and “catalytic activity” included the highest number of DEGs relative to other subcategories and IAV+SP contained the most DEGs than the other groups. In the three categories, ‘‘biological process” is dominant compared to cellular processes and metabolic processes (Figure 7).

**Figure 8:**
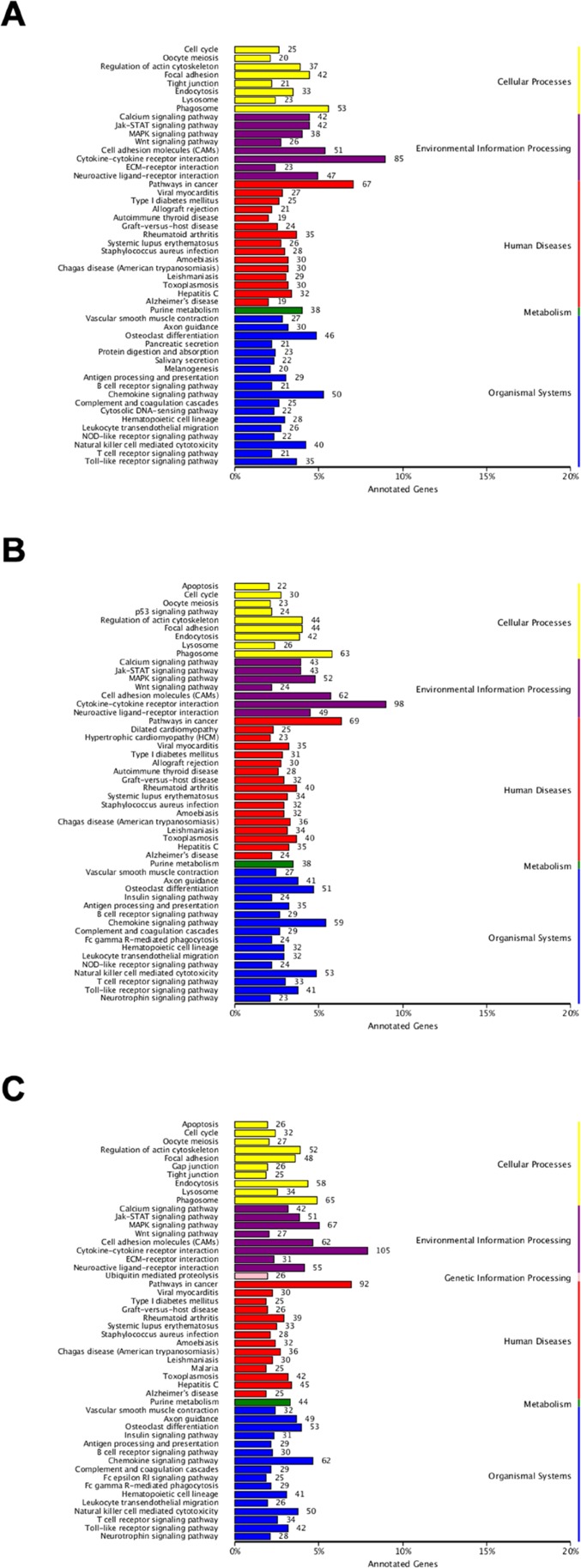
KEGG pathway classification of identified genes The abscissa represents the annotated genes in KEGG database; the ordinate represents Categories in KEGG database. (**A**) KEGG pathway classification of SP. (**B**) KEGG pathway classification of IAV. (**C**) KEGG pathway classification of IAV+SP.

**Figure 9:**
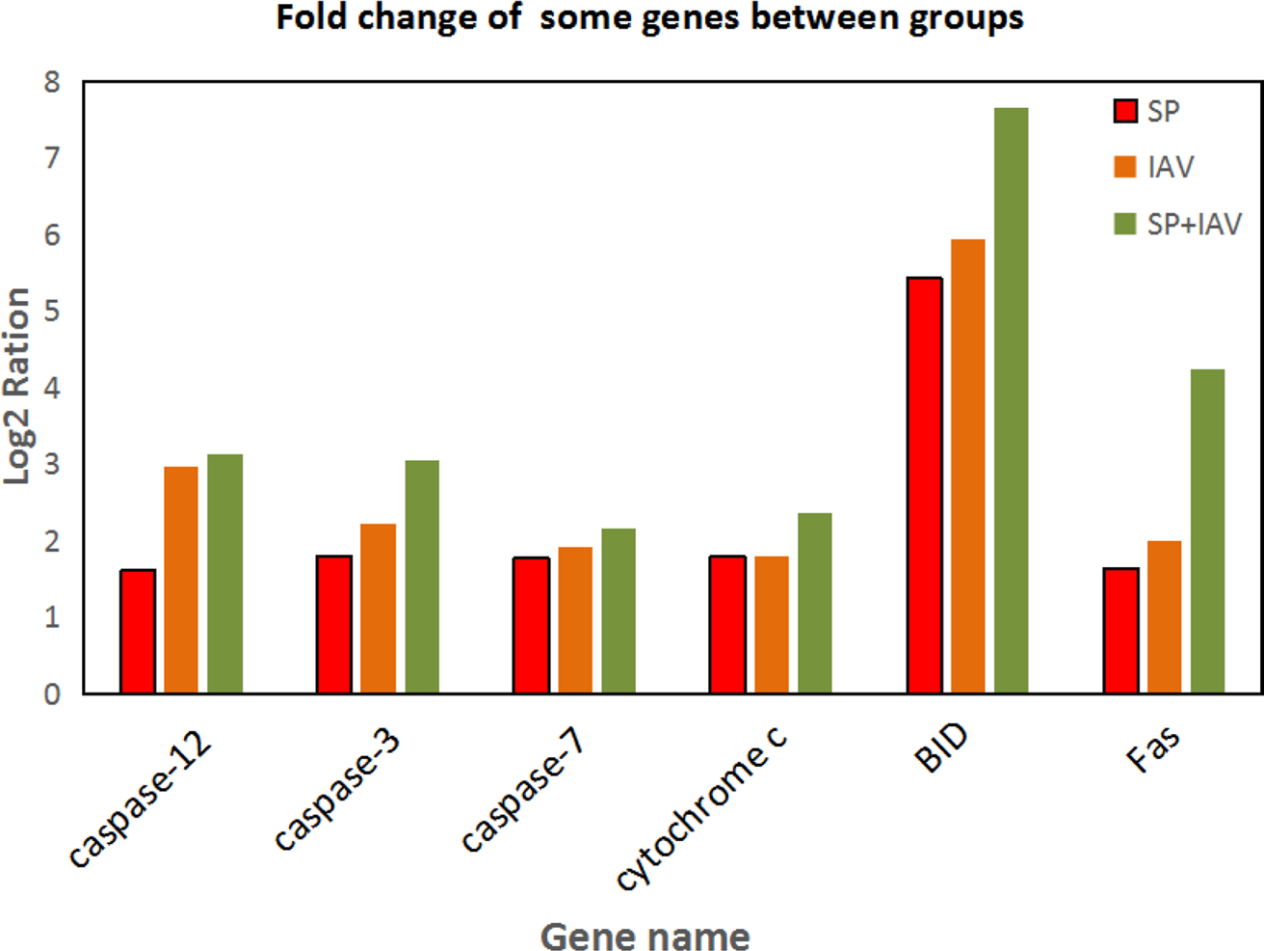
Fold change of some genes which are closely related to apoptosis

To further understand biological functions of the identified genes and classify their functional annotation, the significantly enriched pathways were identified by comparing them to the KEGG database (Figure 8). When comparing Control vs SP, 946 DEGs were annotated to 206 metabolic pathways. Among these pathways, the “Cytokine-cytokine receptor interaction” included the most DEGs (85, ko04060), followed by the “Pathways in cancer” (67, ko05200), “Phagosome” (53, ko04145), “Cell adhesion molecules” (51, ko04514) and “Chemokine signaling pathway” (50, ko04062) etc. The comparison of Control vs IAV was annotated 1089 DEGs to 211 pathways. Most of these DEGs were clustered in the “Cytokine-cytokine receptor interaction” category (98), followed by the “Pathways in cancer” (69), “Phagosome” (63), “Cell adhesion molecules” (62) and “Chemokine signaling pathway” (59) etc. As to Control vs IAV+SP, 1329 DEGs were annotated to 219 pathways. “Cytokine-cytokine receptor interaction” (105) was also annotated the most DEGs, then “Pathways in cancer” (92), “MAPK signaling pathway” (67, ko04010), “Phagosome” (65) and “Chemokine signaling pathway” (62) etc. Some pathways closely related to infection-induced damage were significantly enriched, such as the “apoptosis”, which warranted to be further study. As shown in Figure 9, some genes which closely related to apoptosis revealed remarkable high expression level in SP+IAV group.

### Identification and verification of DEGs

**Figure 10:**
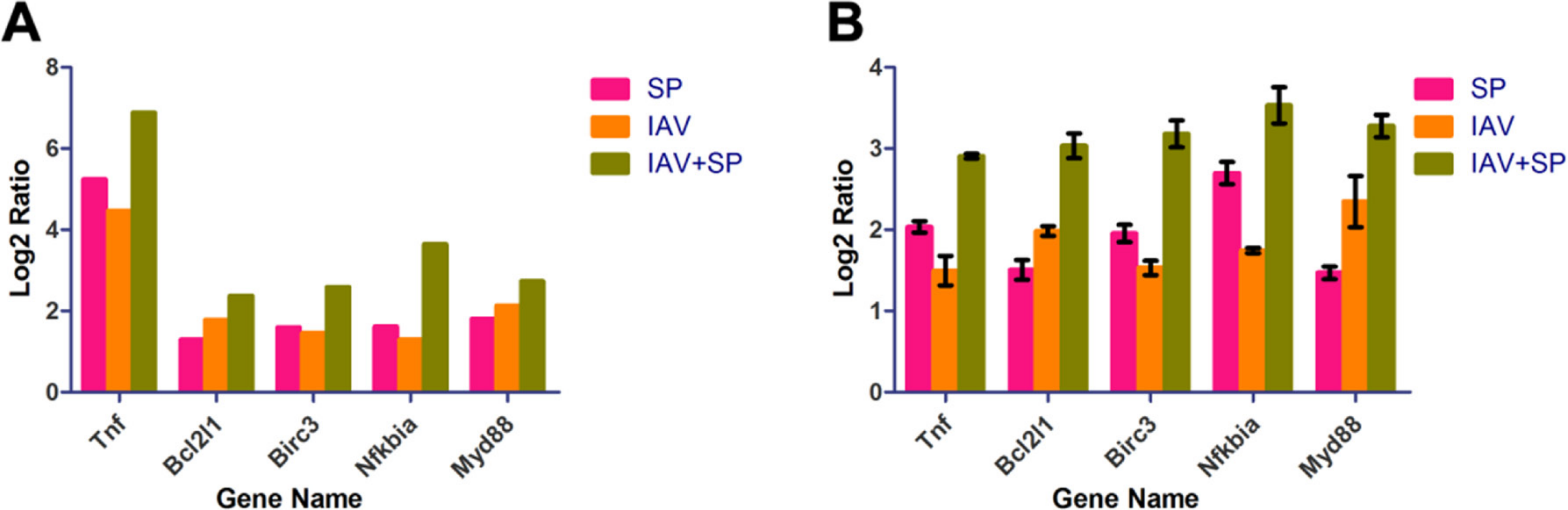
RT-PCR analysis of 5 randomly selected unigenes (**A**) Gene expression data for DGE analysis. The fold changes of the genes were calculated as the log2 vaule of SP/Control, IAV/Control and IAV+SP/Control and shown on the y-axis. (**B**) The qRT-PCR analysis of gene expression data. Expression ratios of these genes in SP, IAV and IAV+SP were compared to Control, respectively.

To validate the expression profiles obtained by RNA-Seq, qRT-PCR was performed on five genes selected at random with high or low expression levels. Expression comparisons were performed between SP and Control, IAV and Control, IAV+SP and Control by qRT-PCR. For all of the genes, the trend in qRT-PCR expression was in agreement with the RNA-Seq data, with only some fold-change discrepancies. This result was consistent with the DGE result, which were considered to be of very high reliability (Figure 10).

## DISCUSSION

It is well known that pneumococcal pneumonia often occurs following primary influenza infection, and secondary pneumococcal pneumonia complicates many severe cases in influenza-infected hosts [1, 12]. Several mechanisms have been suggested to explain the enhanced susceptibility to secondary pneumococcal infections after primary influenza infection. Studies have considered that influenza-mediated lung damage contributed to enhanced susceptibility to secondary bacterial infections [13, 14]. Previous studies showed that apoptosis played a role in influenza virus pathogenesis and in causing cell death and tissue damage during numerous pathogenic responses [15–17].

**Figure 11:**
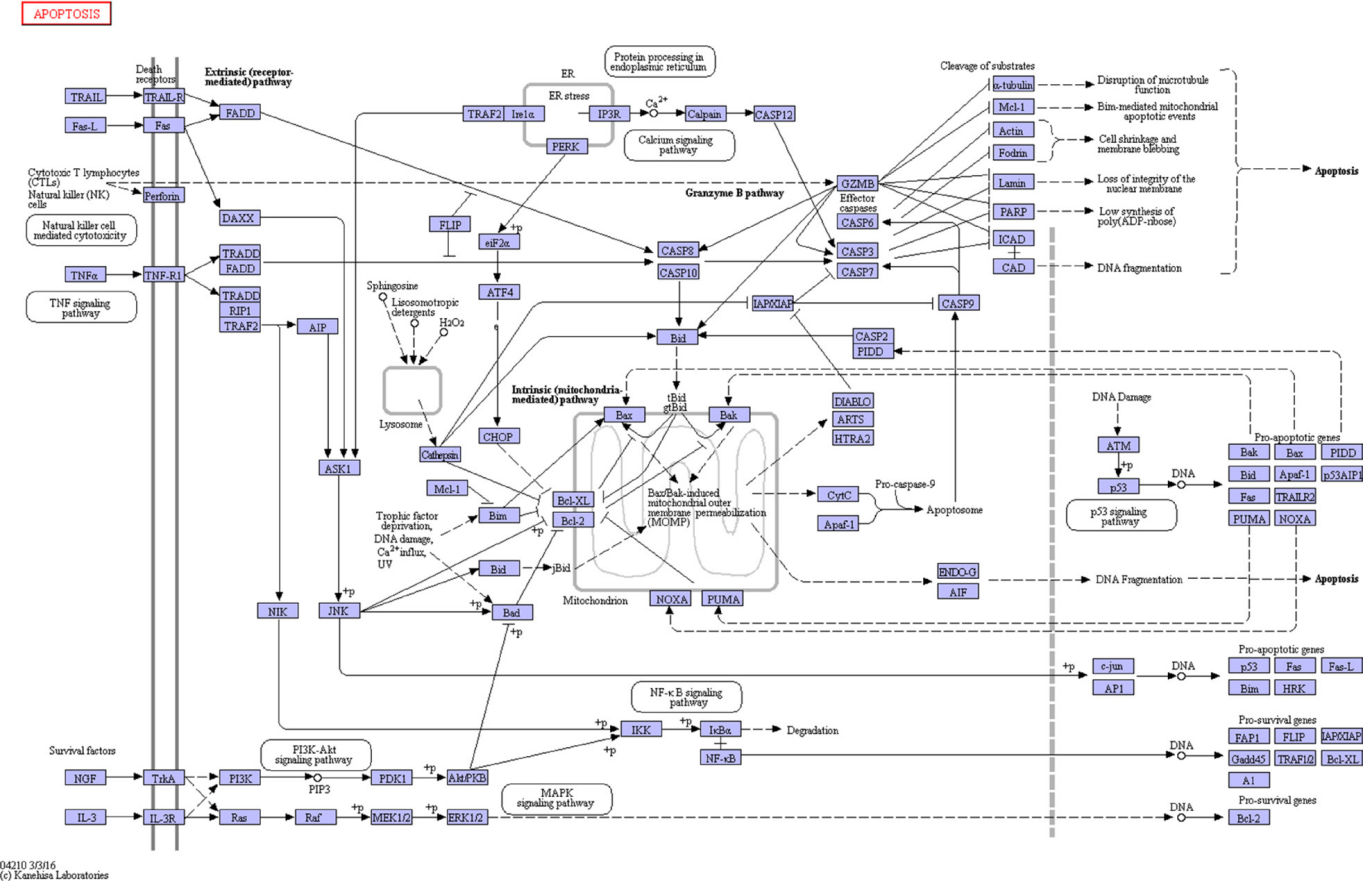
The KEGG pathway of apoptosis

Transduction pathways play crucial roles in the development of infection, participating in key steps in multiple pathways that link extracellular stimuli to intracellular signals [18]. In this study, the apoptosis pathway was significantly differentially activated in particular, which directed and integrated a complex network that was involved in activation of kinases, thereby controlling cell apoptosis and leading lung tissue damage (Figure 11). Alveolar epithelial apoptosis was described as a common feature of acute lung injury caused by direct or indirect factors such as pneumonia, aspiration, sepsis, or trauma [19, 20]. Apoptosis was described to occur in the lungs of patients with acute lung injury [21, 22]. Apoptosis is a regulated form of cell death that induced in several ways, including the death receptor pathway, the mitochondrial pathway, and ER stress-mediated apoptosis [23, 24]. Apoptosis pathway was significantly up-regulated in mice lung due to the infection. In death receptor pathway, upon exogenous apoptotic stimulation, extracellular signals were transmitted into cells through the cell membrane death receptor Fas (fold change of *Fas* : SP=1.64, IAV=2.00, IAV+SP=4.25), which then mediated apoptosis through caspase-8 with subsequent cleavage of procaspase-3 and resulting apoptosis [25]. Caspase-8 was activated and promoted cell apoptosis through the activation of caspase-3, caspase-6, and caspase-7. However, in the mitochondrial pathway, caspase-8 could also cleave Bid (fold change of *Bid* : SP=5.43, IAV=5.93, IAV+SP=7.66) and translate activated Bid [26, 27]. Bid is a member of BH3-only protein that links death receptor crosslinking to pro-apoptotic events at mitochondria. In its uncleaved form, Bid is inactive as an apoptosis inducer. Following death receptor stimulation, Bid is cleaved by caspase-8, resulting in the generation of tBid [28]. Gene tBid is capable of inducing mitochondrial outer membrane permeabilization in cells. Its translocation from the cytoplasm to the mitochondria triggers the release of cytochrome c (fold change of *cytochrome c*: SP=1.80, IAV=1.79, IAV+SP= 2.36) from the mitochondria, which in the cytoplasm triggers the cascade of caspase-9 and caspase-3 activation, causing mitochondrial pathway-regulated apoptosis [26, 27, 29]. Gene caspase-12 (fold change of *caspase-12*: SP=1.61, IAV=2.98, IAV+SP=3.13) is cleaved and specifically activated only during ER stress, and is located on the cytoplasmic side of the ER [30, 31]. It has been reported that caspase-12 is the first ER associated member of the caspase family [32]. During ER stress-induced apoptosis cascade, activation of ER resident caspase-12 will further trigger the activation of cytoplasmic caspase-3(fold change of *caspase-3*: SP=1.80, IAV=2.23, IAV+SP=3.06) and, finally, trigger cellular apoptosis [33]. Caspases are a family of related proteases playing several important functions in apoptosis. They are essential to completion of apoptosis, and are activated in a cascade leading to rapid disablement of key cell structural proteins, chromatin condensation and DNA fragmentation, cell shrinkage and blebbing [34]. Both caspase-3 and caspase-7 (fold change of *caspase-7*: SP=1.77, IAV=1.92, IAV+SP=2.16) are effector caspases performing downstream execution steps of apoptosis by cleaving important cellular substrates [35]. Caspase-7 is highly related to Caspase-3, and these two caspases are activated during the death receptor pathway, mitochondrial pathway, and ER stress-mediated apoptosis [36, 37].

## CONCLUSIONS

To our knowledge, our work represents the first report of the utilization of the next generation sequencing technique DGE for the study of apoptosis in mice when infected with SP , IAV or IAV+SP, with the motality rate was 20%, 35%, 100%, respectively. When identified DEGs inducing apotosis involved in apoptosis pathway, the results demonstrated that the extent of fold change ranked the highest in IAV+SP group, followed by IAV group, then the SP group, which might be coincident with the trend of mortality rate.

## MATERIALS AND METHODS

### Infection model and sample collection

Influenza virus A/PR8 strain (H1N1) was grown in allantoic cavities of 10-day-old fertile chicken eggs for 3 days at 35°C, and the produced virus was stored at −80°C until infection experiments. *Streptococcus pneumoniae* (ATCC6303, a type 3 encapsulated strain, was grown on Columbia Agar plates supplemented with 5% (vol/vol) sheep blood. Female BALB/c ByJ mice (8–10 weeks old; Dashuo Company) were subjected to 4 treatments: PBS(Control), SP, IAV, and IAV+SP (*n* = 20 for each group). SP with the concentration of 1.5 × 105 cfu and IAV of 1 × 103 TCID 50 were diluted in sterile PBS and administered intranasally in a volume of 100 mL (50 mL/nostril) to anesthetized mice. Mice were infected with either IAV or PBS and then challenged 5 days later with *S. pneumoniae*. Mice were monitored at least daily for illness and mortality and then euthanized at the seventh day after the first infection. Lung tissues were collected and placed into 500 mL of sterile PBS. Lung homogenates were used directly for bacterial cultures, or were spun at 10 g for 5 min to collect the supernatants for determination of virus titers. Lung tissues were also collected and stored in liquid nitrogen until used for other purposes. The animal experiments were approved by the Institutional Animal Care and Use Committee of Sichuan University.

### RNA isolation, preparation of library for DGE

Following the manufacturer's protocol, total RNA of each sample was extracted by use of RNAiso Plus reagent (TaKaRa), then purified with absolute alcohol and treated with DNase I. The quality and quantity of both DNA and RNA were checked by gel electrophoresis and the DW-K5500 spectrophotometer (DRAWELL). By use of poly-T oligo attached magnetic beads (Invitrogen), approximately 10 μg RNA of each sample was employed to separate Poly (A) mRNA. After purification, by use of divalent cations through elevated temperature, the mRNA was split into small fragments. According to the manufacturer's protocol of the mRNA-Seq sample preparation kit (Illumina), the RNA fragments were reverse-transcribed which were then used to create the cDNA library. In the single-end libraries, the average insert size was 180bp–200 bp.Then following the vendor's recommended protocol, we operated single-end sequencing (50 bp) on the Illumina Hiseq2500. Raw data presented in this publication have been deposited in the NCBI Short Read Archive.

### Data processing and differentially expressed gene analysis

In order to obtain clean reads, raw reads are processed by cutting off the 3′-adaptor sequence and removing low-quality tags and tags that were too short or those with a single copy number. Then the clean reads were retained and mapped to the reference genome at ftp://ftp.ensembl.org/pub/release-78/fasta/mus_musculus/. Data has been deposited to NCBI and included the accession number (SRP089851).

### Analyzing of differential gene expression

Transcript level of each expressed gene was calculated and normalized to FPKM (the number of fragments per kilo base of exon per million fragments mapped). By using the rigorous algorithm method [38], the detection of differential gene expressions across samples was performed. DESeq software was applied to analyze differential gene expression in biological samples which was used to calculate and compare the differences of gene expression profile between virus or bacteria-treatment groups and control group. In our study, we had three biological repeats for each group and the correlation of the detected number of counts between parallel libraries was assessed statistically by calculating the Pearson correlation. The *P* value threshold in multiple tests and analyses was determined by False discovery rate (FDR). Genes with FDR < 0.01 and log2 ratio ≥ 1 were considered the significant differentially expressed genes (DEGs). The possible functions of DEGs was determined by GO classification system. To further identify significantly enriched pathways, pathway enrichment analysis was performed by using the Kyoto Encyclopedia of Genes and Genomes (KEGG) database.

### Quantitative reverse transcription-polymerase chain reaction (qRT-PCR)

Total RNA was extracted from lung tissues using RNAiso Plus reagent (TaKaRa) and reverse transcribed into complementary DNA (cDNA) using the PrimeScript™ RT reagent Kit (TaKaRa) according to the manufacturer's recommendations. Real-time PCR was performed using SYBR Premix Ex Taq™ (TaKaRa) in the CFX96^™^ Real-Time System (Bio-Rad, USA). The conditions of thermal cycle were 95°C for denaturation in 3 min, then 40 cycles with 95°C in 10 s, followed by 58°C for annealing in 30 s, and then an extension. Each gene was repeated in three times. The primers for qRT-PCR were listed in Table 2. Actin was used as a reference to eliminate bias among samples, and qRT-PCR results were converted and calculated via the 2-ΔΔCt method [39].

**Table 2:** Primers used in qRT-PCR for DGE validation

Gene	Forward primer (5′-3′)	Reverse primer (5′-3′)
Tnf	TCTTCTCATTCCTGCTTGTGG	TACAGGCTTGTCACTCGAA
Bcl2l1	TGGAAGAGAATCGCTAAACACA	TAGGAGAGAAAGTCGACCAC
Birc3	AAAGGCCAAGAATTTGTCAGC	ATCATGACGACATCTTCCGAAC
Nfkbia	ACACGGAGTCAGAATTCACA	GAATCACCCCAGTAAAATGCC
Myd88	AAACGCCGGAACTTTTCGATG	TCGCTTCTGTTGGACACCT
Actb	ATCCGTAAAGACCTCTATGCC	ACACAGAGTACTTGCGCTCA
